# Residue Depletion of Enrofloxacin and Doxycycline in Eggs of Chinese White-Feathered Quail After Administration via Drinking Water

**DOI:** 10.3390/vetsci13090917

**Published:** 2026-09-07

**Authors:** Yabin Zhang, Xudong Fu, Songyan Liu, Wei Lyu, Lining He, Rui Song, Mengxue Zhang, Zhiheng Wang, Yun Li, Jianhua Qin

**Affiliations:** 1 College of Veterinary Medicine, Hebei Agricultural University, Baoding 071000, China; zhangyabin44@163.com; 2 Shijiazhuang Livestock Product and Veterinary Drug Feed Quality Testing Center, Shijiazhuang 050000, China; 3 Shijiazhuang Luancheng District Agricultural Product Quality Monitoring Station, Shijiazhuang 050000, China

**Keywords:** quail egg, enrofloxacin, doxycycline, veterinary drug residues, residue elimination, withdrawal time

## Abstract

The objective of this study was to examine the depletion kinetics of enrofloxacin and doxycycline in quail eggs and develop suitable withdrawal times. Four hundred healthy laying Chinese White-feathered quails were randomly assigned to five groups (*n* = 80 each): a control group and two dose groups (high and low) for each drug. The doses were set at the maximum dose recommended for chickens and at half of that dose, according to the Chinese Veterinary Pharmacopeia. Both drugs were administered continuously for 5 days via drinking water. Eggs were collected at different time points during the administration period and after withdrawal, until residues fell below the detection limit. During the administration period, residues in quail eggs increased progressively, with the highest accumulation found in albumen. After drug withdrawal, the elimination patterns were similar across the dose levels. Residues in albumen declined more rapidly than in yolk. For both drugs, residue concentrations declined rapidly during the initial period after withdrawal and more slowly thereafter. Using the whole-egg residue data and the Chinese national standard (GB 31650.1-2022) maximum residue limits (MRLs) of 10 µg/kg, the withdrawal times were calculated as follows: for enrofloxacin, 10.75 days (0.15 g/L) and 9.03 days (0.075 g/L); for doxycycline, 25.56 days (0.3 g/L) and 21.30 days (0.15 g/L). These results provide valuable data for residue monitoring and risk management, and offer a scientific reference for food safety assurance of quail eggs.

## Simple Summary

Veterinary drug residues are frequently detected in quail eggs. These residues are considered a threat to consumer health and an obstacle to the sustainable development of the quail industry. Therefore, it is necessary to evaluate the elimination patterns of these residues in quail eggs and to determine the withdrawal periods. However, limited studies are available on quail. The present study was designed to investigate the depletion kinetics of two high-risk veterinary drugs, enrofloxacin and doxycycline, in quail eggs. The withdrawal periods were established based on the maximum residue limits (MRLs). When the Chinese national standard MRLs of 10 μg/kg were applied, the withdrawal times for enrofloxacin were estimated at 10.75 days for the 0.15 g/L dose group and 9.03 days for the 0.075 g/L group. For doxycycline, the corresponding values were calculated as 25.56 days (0.3 g/L) and 21.30 days (0.15 g/L). Based on these findings, this study provides essential residue depletion data to support the control of veterinary drug residues in quail eggs, and serves as a technical reference for quail production.

## 1. Introduction

Quail eggs, a traditional specialty poultry product, have gained increasing prominence in human diets owing to their rich nutritional profile and distinctive flavor. They rank third in global production among poultry eggs, after chicken and duck eggs [1]. According to industry statistics, the global quail egg market reached approximately USD 2.002 billion in 2025, with production concentrated mainly in Asia [2]. Within this region, China has emerged as the world’s largest producer and consumer. The commercial quail farming population in China exceeded 500 million birds in 2024 [3]. However, the rapid expansion of the quail industry has raised food safety concerns that cannot be overlooked. Our survey of poultry eggs collected from Chinese e-commerce platforms showed that the detection and exceedance rates of veterinary drug residues in quail eggs (6/37, 16.2% and 5/37, 13.5%, respectively) were higher than those in chicken eggs (4/68, 5.9% and 3/68, 4.4%, respectively) [4]. Similar observations have also been reported in other studies [5,6]. These findings indicate that food safety risks associated with quail egg residues deserve greater attention. Notably, enrofloxacin (ENR) and doxycycline (DOX) are the compounds frequently identified in quail eggs [7]. Such residues may cause multiple adverse health risks to consumers, including allergic reactions, immunopathological damage, hepatotoxicity, reproductive disorders, and potential mutagenic and carcinogenic effects. Moreover, they may promote the dissemination of antimicrobial resistance [8,9].

ENR and DOX belong to the fluoroquinolone and tetracycline antimicrobials, respectively, and are widely applied for the treatment of bacterial diseases in poultry. Given their residue hazards, many countries and regions have imposed strict restrictions on their use in laying poultry. The United States (U.S.) Food and Drug Administration (FDA) withdrew approval for enrofloxacin use in poultry in 2005 and has not approved any doxycycline product for use in laying poultry to date. Both China and the European Union (EU) have prohibited the use of ENR and DOX in laying birds [10,11,12]. Regarding maximum residue limits (MRLs), the U.S. and the EU have adopted a zero-tolerance policy for residues of these drugs or their metabolites in poultry eggs, while China has set an MRL of 10 μg/kg [13]. Importantly, the prohibitions implemented in China and the EU apply only to the laying period. Administration of these drugs in poultry during the pre-laying (rearing) phase is not explicitly forbidden. Several EU member states (e.g., the Netherlands, Germany, and Spain) have issued specific label recommendations for pre-laying medication. For example, a 14-day withdrawal period for ENR and a 4-week withdrawal period for DOX are recommended prior to the onset of lay [14]. This management practice, however, is conditional upon effective drug clearance before the onset of lay. Whether complete elimination can be achieved depends on the depletion kinetics of these compounds in the target avian species. If drug clearance is incomplete, residues may persist beyond the onset of lay and be continuously transferred into eggs. This inference is indirectly supported by our field observations. In our traceback investigations conducted by our group on quail egg samples positive for ENR or DOX residues, no evidence of medication during the laying period was found. These results suggest that the residue most likely arises not from illegal use during the laying period, but from therapeutic treatment administered prior to lay. However, direct validation of this hypothesis remains unattainable owing to the current lack of depletion data for ENR and DOX in quail eggs.

Given this research gap, accurate determination of withdrawal intervals for safe egg consumption requires species-specific residue depletion data. Such data are available for chickens but remain scarce for quails. In practical production, the quail industry lacks standardized medication guidelines [15], so producers often rely on chicken-derived experience for breeding and medication management. One previous study on florfenicol has confirmed pharmacokinetic differences between quails and chickens. Therefore, direct extrapolation of research findings from chickens to quails is inadvisable [16], as this practice may pose residue-related risks. These observations underscore the need for dedicated residue depletion studies of ENR and DOX in quails.

Although ENR and DOX may be administered prior to lay, residue depletion investigations cannot be performed in the pre-laying period, owing to the absence of eggs for sampling. Therefore, this study employed peak-laying quails as an experimental model to simulate residue carry-over into lay, instead of performing pre-laying administration. Using this model, we systematically investigated the depletion kinetics of ENR and DOX in quail eggs after drinking water administration. The study objectives were to characterize the depletion profiles, determine withdrawal periods and safe medication windows, and provide scientific data for rational drug use and safe production in quails.

## 2. Materials and Methods

### 2.1. Drugs and Reagents

Experimental drugs: 10% enrofloxacin soluble powder, batch number 20241102, and 10% doxycycline hydrochloride soluble powder, batch number 20241001, Jiangxi Youdao Pharmaceutical Co., Ltd. (Jiujiang, China).

Reagents: Acetonitrile (chromatographic grade), formic acid and ammonium acetate (Thermo Fisher Scientific, Waltham, MA, USA); Na_2_EDTA, citric acid, and sodium citrate (analytical grade, Tianjin Yongda Reagent Co., Ltd., Tianjin, China); distilled water (Watsons, Guangzhou, China); mass spectrometry calibration solutions (positive ion mode, AB Sciex, Framingham, MA, USA).

Reference standards: Enrofloxacin, ciprofloxacin, and doxycycline (Tianjin Alta Technology Co., Ltd., Tianjin, China).

### 2.2. Experimental Animals and Design

#### 2.2.1. Experimental Animals

The animal experiment was approved by the Laboratory Animal Ethics Committee of Hebei Agricultural University (approval No. 2021049; approval date: 1 March 2021). A total of 400 healthy Chinese White-feathered quails at 180 days of age in the peak laying period were selected for this study. Pre-laying quails cannot provide egg samples for residue testing, whereas peak-laying quails have fully mature reproductive organs and can provide continuous egg samples, which enables generation of conservative risk-assessment data for this preliminary study. Prior to the formal experiment, all birds were acclimatized for one week. During this period, the quails had free access to feed and water. An antibiotic-free diet (layer pellet feed for quails, produced by Shijiazhuang Fengda Animal Husbandry Co., Ltd., Shijiazhuang, China) was provided. Feed samples were collected and analyzed to confirm the absence of the target analytes. Throughout the experimental period, the quails were housed in cages under stable environmental temperature and humidity. During the acclimatization period, daily monitoring was performed on physiological indicators (including feed and water intake, respiratory condition, mental status, egg production, and excreta), and egg samples were collected for analysis.

#### 2.2.2. Experimental Design

During the formal experimental period, 400 quails were randomly divided into five groups, with 80 birds in each group. The experimental design comprised one control group, two ENR-treatment groups, and two DOX-treatment groups. For each drug, a high-dose and a low-dose group were established, with four replicates per group and 20 quails per replicate. The grouping and design of the residue depletion experiment are presented in Table 1.

#### 2.2.3. Administration Method

As no specific dosage recommendations for enrofloxacin and doxycycline are available for quails, the administered doses were determined according to the relevant provisions for chickens stipulated in the Veterinary Pharmacopeia of the People’s Republic of China [17]. The high-dose groups received the maximum recommended dose for chickens as specified in the Pharmacopeia, while the low-dose groups received half of this dose. The drugs were administered via drinking water, with ad libitum access to medicated water throughout the treatment period. Under group-housing and free-drinking conditions, individual water intake of quails could not be recorded; therefore, water consumption and individual drug intake were not monitored in this study. Although individual intake may vary under free-drinking conditions, eggs from each replicate were pooled into a single sample for residue analysis to mitigate the influence of this variation. The duration of medication was set at 5 days, which corresponds to the maximum recommended treatment period in the Pharmacopeia. During the medication period, the quails were fed an antibiotic-free diet with free access to feed, and their physiological status was monitored daily.

#### 2.2.4. Sample Collection

During drug administration, eggs were collected daily from all experimental groups. For the enrofloxacin groups, eggs were sampled on days 1, 2, 3, 5, 7, 9, 12, 15, and 18 after drug withdrawal. For the doxycycline groups, eggs were sampled on days 1, 2, 3, 5, 9, 15, 21, 28, 35, 42, 49, and 56 after drug withdrawal. The collected eggs were divided into two portions: one portion was used to prepare whole-egg samples, and the other portion was separated into albumen and yolk samples. After homogenization, all samples were placed in clean sampling vials, labeled with identification numbers, and stored at −20 °C until analysis.

### 2.3. Sample Preparation

Target analytes: For the ENR groups, ENR and its metabolite ciprofloxacin (CIP) were determined. For the DOX groups, DOX was determined.

Sample preparation: Drug extraction from eggs was performed as previously described by Chen H. [18], with some modifications. Samples of 2.00 g (±0.01 g) of homogenized yolk, albumen, or whole egg were weighed and placed into the centrifuge tubes. Then, 2 mL of 0.05 mol/L Na_2_EDTA solution was added, and the mixture was vortexed for 1 min. Subsequently, 8 mL of 0.1% formic acid in acetonitrile was added, and the sample was shaken for 10 min, sonicated for 20 min, and centrifuged at 10,000 rpm for 10 min at 4 °C. The supernatant was transferred to a fresh centrifuge tube. The extraction was repeated once, and the supernatants were combined. For yolk samples, an additional defatting step was performed: 20 mL of acetonitrile-saturated n-hexane was added to the combined supernatant, vortexed for 1 min, and centrifuged at 10,000 r/min for 2 min at 4 °C. The n-hexane layer was discarded, and the lower layer was retained as the test solution. Purification: A 6 mL aliquot of the test solution was passed through an Oasis PRiME-HLB cartridge, and the eluate was collected. The eluate (5 mL) was evaporated to dryness under nitrogen at 40 °C. The residue was reconstituted in 0.5 mL of 5% acetonitrile in water containing 0.1% formic acid, filtered through a 0.22 µm polyvinylidene fluoride membrane, and analyzed by LC-MS/MS.

### 2.4. Liquid Chromatography–Mass Spectrometry Conditions

Chromatographic separation was performed on a Prominence LC-30A high-performance liquid chromatography system (Shimadzu, Kyoto, Japan) coupled with a 5500 QTRAP triple quadrupole mass spectrometer (AB Sciex, Framingham, MA, USA).

Chromatographic conditions: Chromatographic column: Kinetex F5 column (100 mm × 3.0 mm i.d., particle size 2.6 μm; Phenomenex, Torrance, CA, USA), which could provide better separation of ENR, CIP, and DOX than conventional C18 columns. Column temperature: 40 °C. Mobile phase: 0.1% formic acid in water (A) and 0.1% formic acid in acetonitrile (B). Flow rate: 0.4 mL/min. Injection volume: 5 µL. Gradient elution conditions: 0~1.00 min, 97% A and 3% B; 1.10 min, 60% A and 40% B; 5.00 min, 10% A and 90% B; 6.00 min, 97% A and 3% B; 7.00 min, stop. A 2 min equilibrium time was allowed between injections for both yolk and albumen samples.

Mass spectrometry conditions: Electrospray ion source (ESI+); positive ion scanning mode; mass spectrometry scanning mode: dynamic multiple reaction monitoring (MRM). The characteristic ion parameters for target analytes are provided in Table 2. Data acquisition and quantitative analysis were performed using Analyst 1.6.2 and MultiQuant 3.0.2, respectively (AB Sciex, Framingham, MA, USA).

### 2.5. Method Validation

This analytical method was validated in accordance with Commission Implementing Regulation (EU) 2021/808 [19] and GB/T 27417-2017 Guidelines for the Validation and Verification of Chemical Analytical Methods [20]. Linearity was evaluated using matrix-matched calibration curves prepared at six concentration levels (2, 5, 10, 20, 50, and 100 μg/kg) by spiking blank matrix with mixed working standard solutions of target analytes. All sample quantitation was performed against these matrix-matched calibration curves. For recovery and precision assessment, blank matrix samples were spiked at three concentration levels (2, 10, and 50 μg/kg). Following extraction and clean-up, samples were analyzed by LC-MS/MS to calculate recovery and precision parameters. Six replicate samples were prepared for each spiking level. Intra-batch and inter-batch coefficients of variation were determined via repeated measurements conducted over three consecutive days. The limit of detection (LOD) and limit of quantification (LOQ) were determined by analyzing spiked blank matrix samples. The LOD was defined as the lowest analyte concentration yielding a chromatographic peak with a signal-to-noise ratio (S/N) of 3, whereas the LOQ corresponded to the concentration giving an S/N of 10.

### 2.6. Data Processing

This study aimed to characterize residue depletion patterns and estimate withdrawal times. Residue concentrations in quail egg samples were analyzed using descriptive statistics and are expressed as mean ± standard deviation (SD).

Withdrawal times were calculated using WT 1.4 software developed by the European Medicines Agency. The software performs linear regression analysis on log-transformed residue data, with the withdrawal time defined as the time point where the upper one-sided 95% tolerance limit of residue concentrations falls below the MRL. Residue data above the LOQ at each time point were selected for regression analysis. As the software allows a maximum of seven time points, the day-15 post-withdrawal data point was excluded from the DOX high-dose group to retain the key early and late withdrawal time points, thereby ensuring adequate characterization of the overall elimination curve. Following data selection, WT 1.4 automatically performed the F-test, Bartlett’s test, and Shapiro–Wilk test to verify the assumptions of linearity, homogeneity of variances, and normality of residuals, respectively, and the results confirmed that all assumptions were satisfied. The corresponding withdrawal times were subsequently determined.

## 3. Results

### 3.1. Method Validation Results

**Figure 1:**
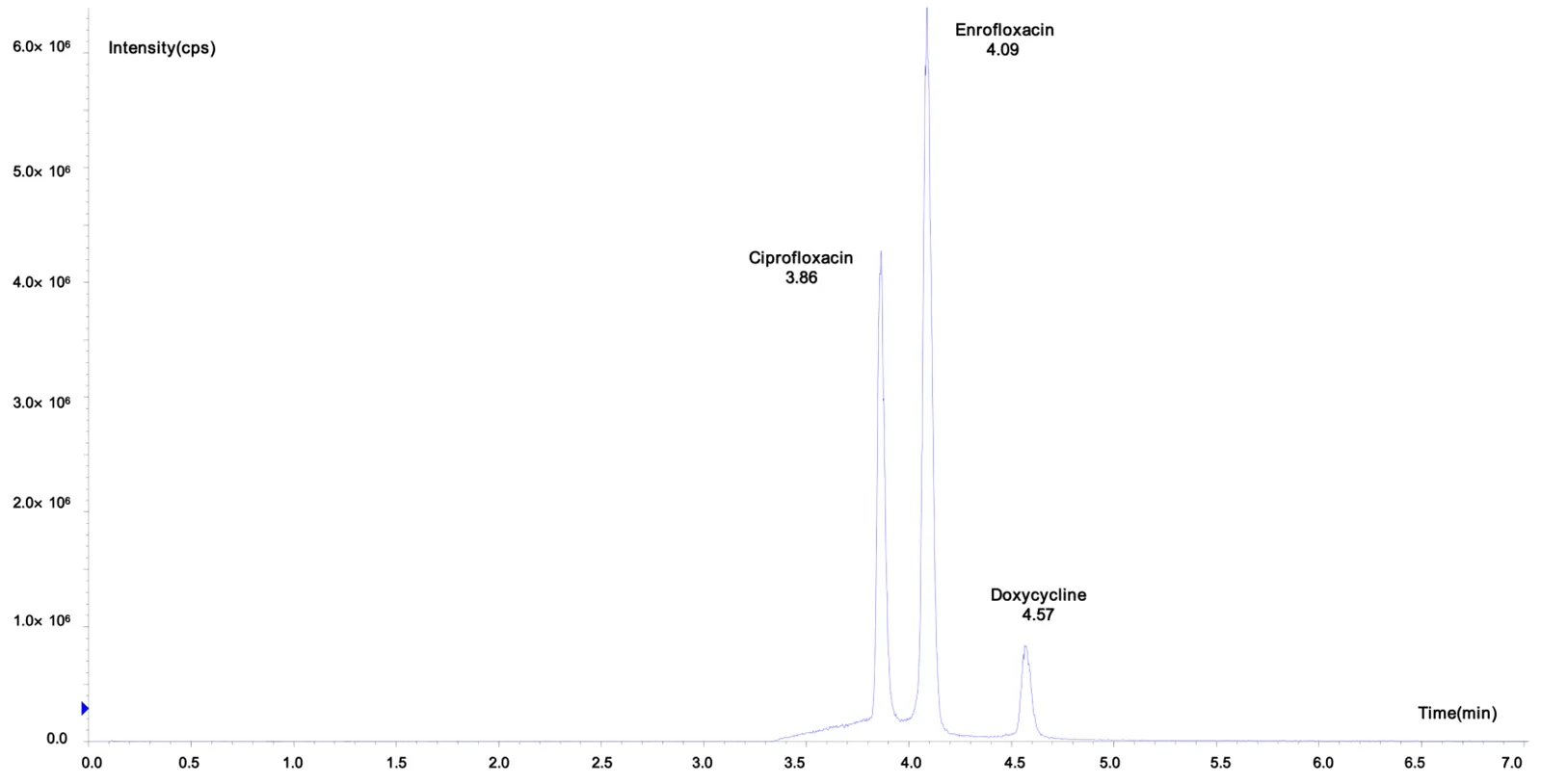
Total ion current chromatogram of three analytes in quail whole egg samples spiked at 20 μg/kg.

Under the established mass spectrometry analysis conditions, the LC-MS/MS method was employed to determine the concentration of the matrix-matched standard solution, and a matrix standard curve was established. All matrix-matched calibration curves exhibited a good linear relationship in the range of 2~100 ng/mL, with a correlation coefficient R^2^ greater than 0.99 (Figures S1–S3). The limit of quantification (LOQ) in each matrix was 2 μg/kg, and the limit of detection (LOD) was 1 μg/kg. The intra-batch coefficient of variation ranged from 4.5% to 9.8%, and the inter-batch coefficient of variation ranged from 4.1% to 9.4%. The average recovery was between 70.35% and 97.65%. A relatively lower recovery of doxycycline was observed in yolk matrix, although all recovery values across whole-egg, albumen, and yolk still fell within the acceptable ranges for EU method validation. The results indicated that the established method for target drugs in quail egg samples met the methodological requirements. The LOQ was five-fold lower than the MRL, and thus the method could be applied to residue elimination studies in quail eggs. The total ion current chromatogram of the target drugs in quail eggs is shown in Figure 1. The detailed results of the methodological evaluation are presented in Table 3.

### 3.2. Elimination Rules of Enrofloxacin and the Main Metabolite in Quail Eggs

**Figure 2:**
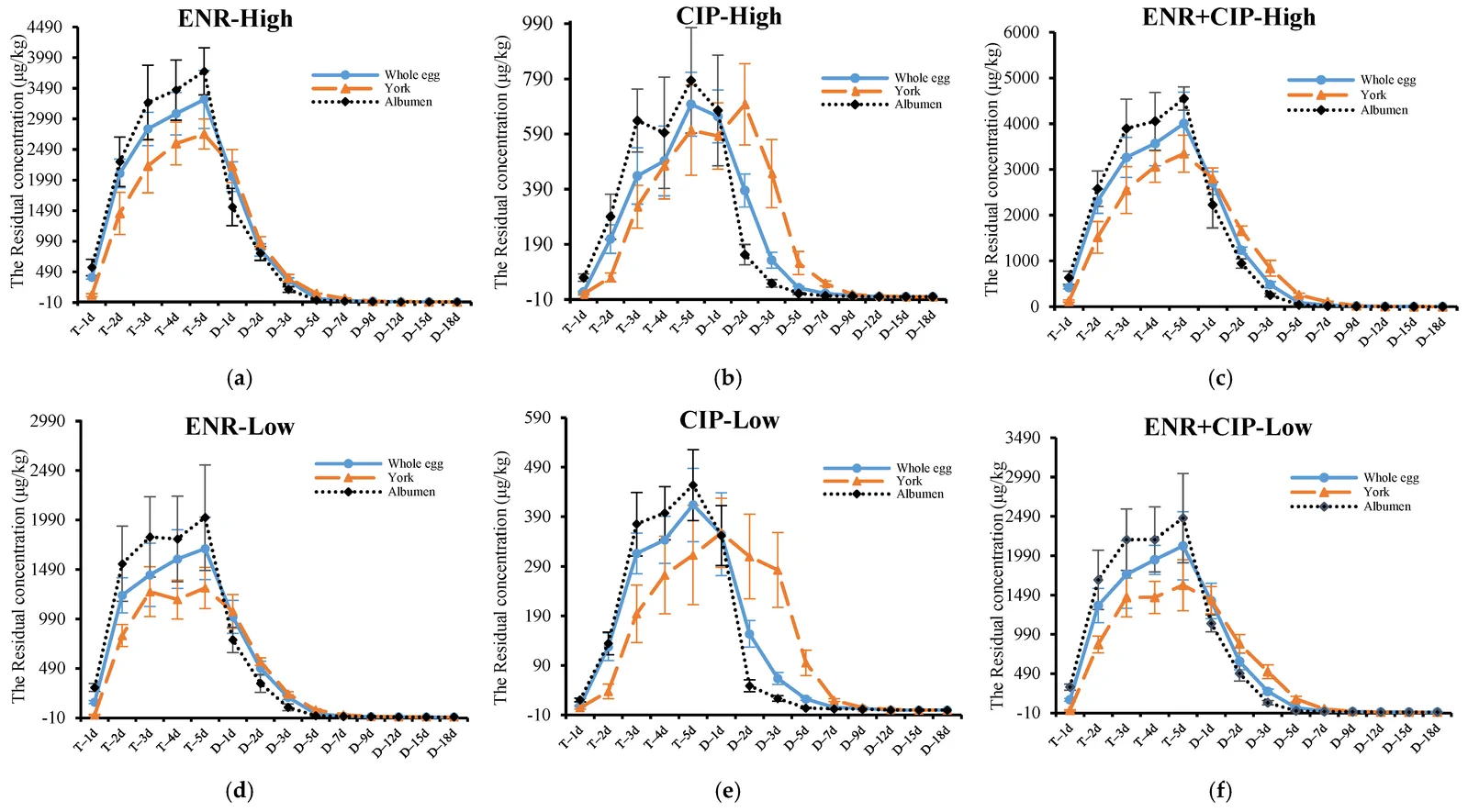
The residue elimination characteristics of enrofloxacin (ENR) and its metabolite, ciprofloxacin (CIP), in quail whole eggs, albumen, and yolks. T–1d denotes the first day of medication treatment, T–2d, T–3d… denote the second day, third day… of treatment; D–1d denotes the first day of withdrawal, D–2d, D–3d… denote the second day, third day… of withdrawal, respectively. (**a**) Elimination curve of ENR in the high-dose group; (**b**) Elimination curve of CIP in the high-dose group; (**c**) Elimination curve of the total residual concentrations of ENR and CIP in the high-dose group; (**d**) Elimination curve of ENR in the low-dose group; (**e**) Elimination curve of CIP in the low-dose group; (**f**) Elimination curve of the total residual concentrations of ENR and CIP in the low-dose group.

In this study, ENR was administered to quails via drinking water at two doses for five consecutive days, and the residue depletion profiles of ENR and its metabolite CIP in quail eggs were determined. As shown in Table S2 and Figure 2, both ENR and CIP were detected in eggs on day 1 post-administration. Residue concentrations rose rapidly on day 2 and then the increase moderated with continued administration, with consistent trends across the two dose groups. ENR residues in whole eggs, albumen, and yolk all peaked on day 5 of administration, with the highest levels found in albumen. On the basis of our observational data, these peak values were approximately 31.24% higher in albumen than in yolk. CIP also showed an overall increasing trend during administration. Its peak level in whole-egg and albumen occurred on day 5, whereas yolk CIP peaked after drug withdrawal: day 1 in the low-dose group and day 2 in the high-dose group, with an approximate lag of 24 h and 48 h relative to yolk ENR peaks. The total residues of ENR and CIP in whole-egg samples peaked on day 5 and were positively associated with administered dose. The maximum value in the high-dose group (4004.46 ± 686.14 μg/kg) was approximately twice that of the low-dose group (2117.39 ± 430.50 μg/kg). Throughout the administration period, ENR residues were higher than CIP, and concentrations of both compounds were greater in albumen compared with whole eggs and yolk. (Table S2 is provided as Supplementary Material).

After drug withdrawal, ENR residues declined rapidly in whole eggs, yolk, and albumen in the early phase. As residual levels decreased over time, the reduction became slow. CIP in albumen also declined rapidly after drug withdrawal. In contrast, yolk CIP kept increasing and reached its peak on day 1 post-withdrawal in the low-dose group and on day 2 post-withdrawal in the high-dose group. This time-lag effect may be attributable to the fact that CIP is the primary metabolite of ENR, which may produce a temporal offset in its appearance and depletion relative to the parent compound. Based on measured concentration-time data, the magnitude of concentration reduction was smaller in yolk than in albumen for both ENR and CIP. Accordingly, residues in yolk remained consistently higher than those in whole-egg and albumen. For whole-egg samples from the low-dose group, the total residues of ENR and CIP fell below the Chinese MRL of 10 μg/kg on day 9 post-withdrawal and became undetectable (below the LOQ) on day 12. In the high-dose group, total residues dropped below the MRL on day 12 and were undetectable on day 15.

**Figure 3:**
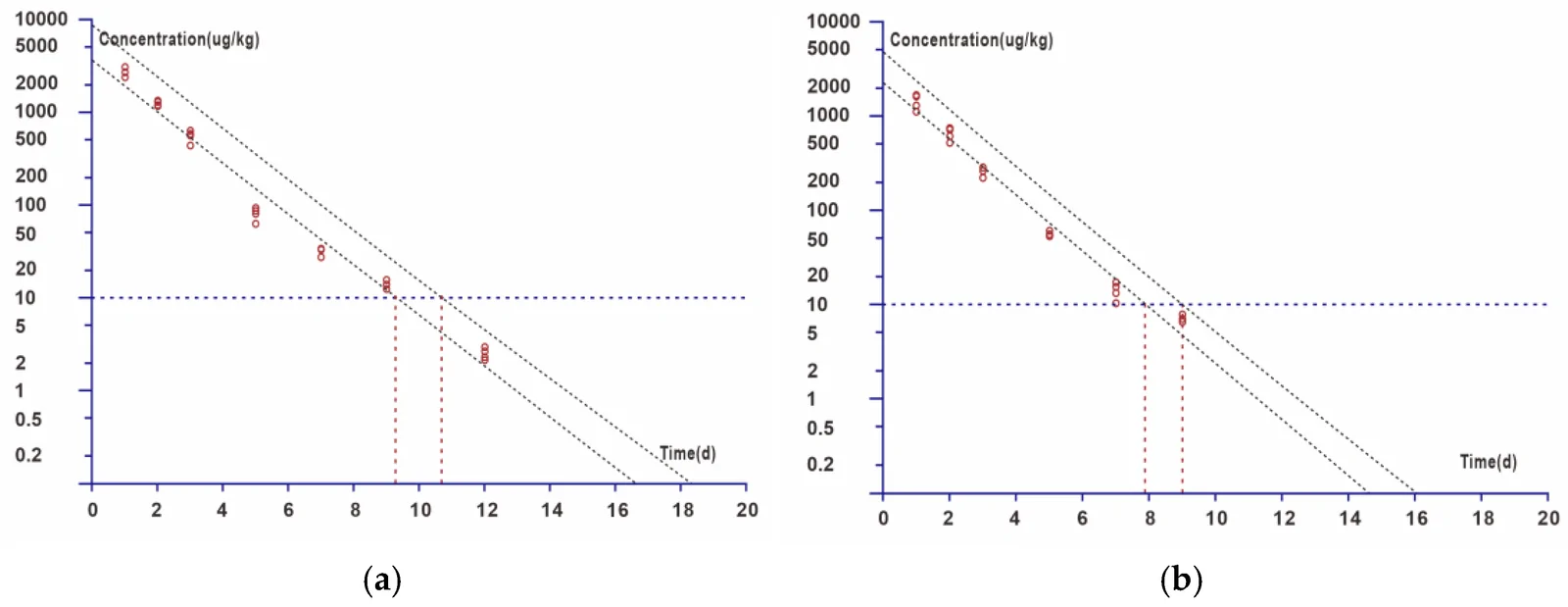
The Calculation results of withdrawal times for enrofloxacin (ENR) in quail whole eggs by WT 1.4 software to keep them below the MRLs (with 95% tolerance limits and 95% confidence intervals). (**a**) The results of the high ENR dose group; (**b**) The results of the low ENR dose group. Each circle represents an individual concentration of drug measured per day. The black lines are the linear regression and 95% tolerance limit with 95% confidence. The blue line represents the maximum residue limit(MRL) 10 μg/kg in GB 31650.1-2022 [13]. The data are calculated based on the total residual amount of ENR and CIP.

To calculate the accurate residue risk periods of ENR in quail eggs, the WT1.4 software was applied for data analysis. Since the MRL for veterinary drug residues in eggs is typically established based on whole-egg residue depletion data, whole eggs were selected for the analysis. Given that ENR is metabolized to CIP and the MRL is expressed as the sum of the parent drug and its metabolites, the residue risk periods were calculated using the total residues of ENR and CIP. According to the provisions of GB 31650.1-2022 regarding the MRL in poultry eggs, linear regression analysis of residue concentrations in quail egg samples was performed using WT1.4 software at a 95% confidence interval. As shown in Figure 3, following continuous drinking water administration for 5 days, the withdrawal times were calculated as 10.75 days for the high-dose ENR group and 9.03 days for the low-dose group.

### 3.3. Elimination Rules of Doxycycline in Quail Eggs

**Figure 4:**
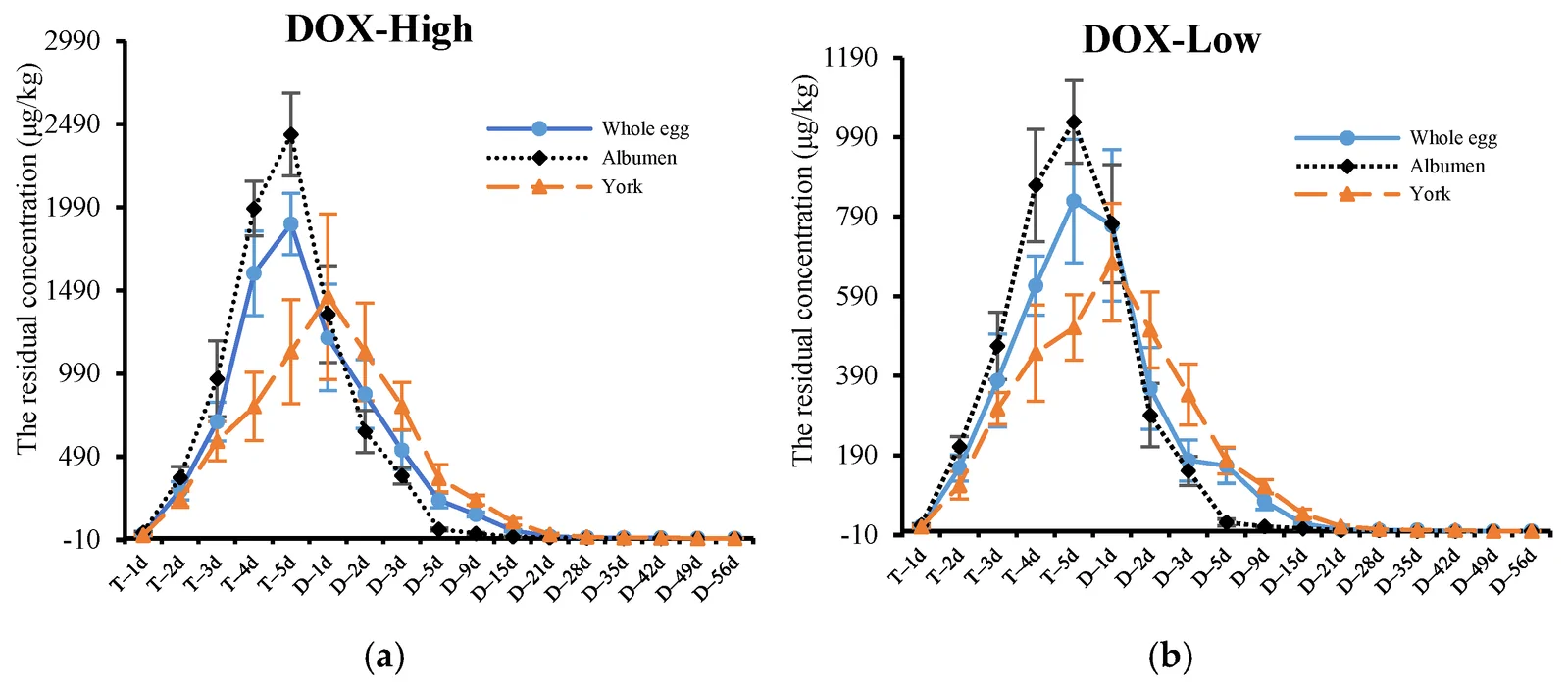
The residue of doxycycline (DOX) in quail whole eggs, albumen, and yolks. T–1d denotes the first day of medication treatment, T–2d, T–3d… denote the second day, third day… of treatment; D–1d denotes the first day of withdrawal, D–2d, D–3d… denote the second day, third day… of withdrawal, respectively. (**a**) The elimination curve of residual levels in quail eggs for the high DOX dose group; (**b**) the elimination curve of residual levels in quail eggs for the low DOX dose group.

The results are presented in Table S3 and Figure 4. DOX was detectable in quail eggs on day 1 post-administration, with residues of 24.01 ± 5.25 μg/kg and 12.28 ± 3.37 μg/kg in the high- and low-dose groups, respectively. From day 2 to day 5 of administration, DOX residues in whole eggs, albumen and yolk continued to increase, with similar trends between the two dose groups. Residues in albumen and whole eggs peaked on day 5 of administration, whereas yolk residues reached their maximum on day 1 post-withdrawal. Throughout the medication period, albumen residues were consistently higher than yolk residues, and the highest and the maximum residues were recorded in albumen. (Table S3 is provided as Supplementary Material).

After withdrawal, DOX residues in albumen declined rapidly. Based on measured concentration-time data, the magnitude of concentration reduction was smaller in yolk than in albumen; from day 2 post-withdrawal onward, yolk residues exceeded those in albumen. Albumen DOX residues fell below the Chinese MRL of 10 μg/kg on day 15 post-withdrawal in both dose groups, and dropped below the LOQ on day 28 (low-dose) and day 35 (high-dose). In yolk, residues in both groups fell below the MRL on day 28 post-withdrawal and below the LOQ on day 42. In whole eggs, residues fell below the MRL on day 21 (low-dose) and day 28 (high-dose) post-withdrawal, respectively, and both groups dropped below the LOQ on day 42.

**Figure 5:**
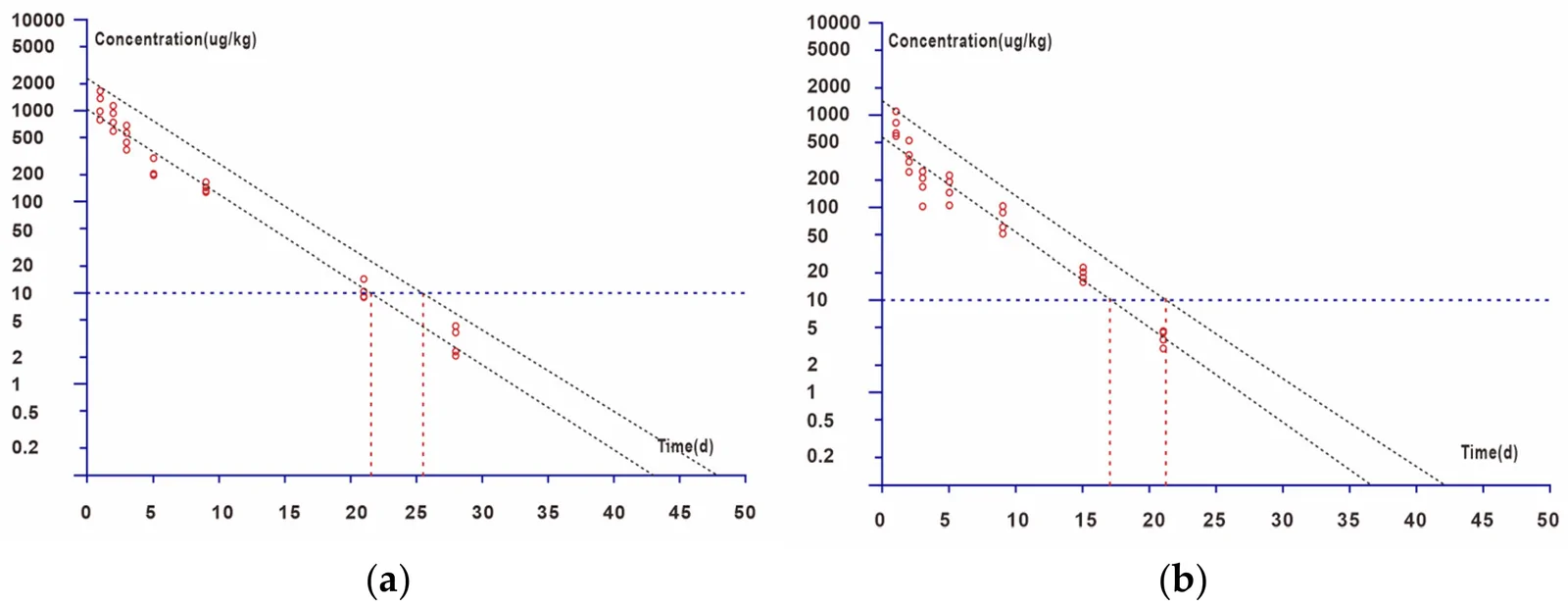
The calculation results of withdrawal times for doxycycline (DOX) in quail whole eggs by WT 1.4 software to keep them below the MRLs (with 95% tolerance limits and 95% confidence intervals). (**a**) The results of the high DOX dose group; (**b**) the results of the low DOX dose group. Each circle represents an individual concentration of drug measured per day. The black lines are the linear regression and 95% tolerance limit with 95% confidence. The blue line represents the maximum residue limit(MRL) 10 μg/kg in GB 31650.1-2022 [13].

According to the MRL provisions of GB 31650.1-2022, with an MRL of 10 μg/kg, linear regression analysis of DOX residue data in quail egg samples was performed using WT1.4 software at a 95% confidence interval. As shown in Figure 5, the withdrawal times were calculated as 25.56 days for the high-dose group and 21.30 days for the low-dose group.

## 4. Discussion

This study systematically investigated the residue depletion profiles of ENR and DOX in eggs of Chinese white-feather quails following drinking water administration. Corresponding withdrawal periods and theoretical safe pre-laying medication windows were calculated to evaluate the clinical applicability of the two antibiotics in egg-type quail production.

Residue concentrations of ENR and DOX were consistently higher in albumen than in yolk throughout the medication period. Albumen residues reached peak levels earlier and declined more rapidly after drug withdrawal, which is consistent with previous references on multiple veterinary drugs in chicken eggs [18]. These phenomena may be associated with multiple factors, including the physicochemical properties of the drug, as well as the formation processes of albumen and yolk. Both ENR and DOX have high water solubility, low lipophilicity, and strong protein-binding ability [21,22], which facilitates accumulation in the water-rich, protein-dominant albumen. In addition, albumen forms rapidly within 2–3 h after ovulation, and its residue levels dynamically correspond to real-time plasma drug concentrations, allowing faster residue saturation and elimination. In contrast, yolk maturation lasts 7–11 days [23], during which continuous drug transfer from plasma to developing yolk prolongs drug retention and slows depletion.

In quail eggs, ENR is mainly present as parent drug and its metabolite CIP, with parent ENR accounting for most total residues. ENR was eliminated more rapidly than CIP, which may be related to the continuous metabolic conversion of ENR to CIP, resulting in a delayed accumulation of CIP in eggs. Accordingly, residue risk assessment relying only on parent ENR may underestimate overall hazards. The total residues of ENR and CIP in whole eggs increased with administration time, peaked on day 5, and showed a positive correlation with dose. After withdrawal, the total residues remained above the MRL (10 μg/kg) until day 9 (high-dose) and day 7 (low-dose), fell below detection limits on day 15 and day 12, giving estimated withdrawal times of 10.75 days and 9.03 days, respectively. Most available ENR residue data originate from laying hen studies, with variable results due to different experimental designs. Pre-laying medication has been reported to induce higher residue levels and slower elimination than laying-period medication [24]. Drug dosage, administration routes, and poultry species are common factors affecting residue profiles [25,26,27]. In this study, medication was performed using drinking water concentrations as recommended by the Chinese Veterinary Pharmacopeia, without monitoring individual water intake, and body-weight-normalized dosages were unavailable. Therefore, it is difficult to make a direct comparison with the studies conducted on chickens. Nevertheless, the present results reliably reflect ENR depletion characteristics in quails under conventional clinical medication.

DOX exhibited longer residue persistence in quail eggs than ENR in our study. As a tetracycline antibiotic with high tissue permeability and plasma protein binding rate [28], DOX forms a stable blood drug reservoir that is gradually released after withdrawal, continuously accumulating in ovarian follicles and extending elimination cycles. DOX residues in whole eggs fell below the MRL (10 μg/kg) on day 28 (high-dose) and day 21 (low-dose), and dropped below the LOQ on day 42, with calculated withdrawal periods of 25.56 days and 21.30 days, respectively. Ou Y.H. [29] reported a 38-day withdrawal period in laying hens under the same medicated water concentration, administration route, and treatment duration, which was longer than the value determined for quails in our study. This indicates that even under consistent experimental conditions, inter-species differences in body weight, water consumption, and physiological metabolism may lead to variations in actual drug intake and in vivo drug behavior, ultimately contributing to differences in residue elimination time. While the studies of Gajda A [30] and Mao Z.Q. [31] in laying hens indicated that drug dose and administration routes also influence DOX elimination, these conventional factors cannot fully explain the observed inter-species result differences. Dose-dependent residual variations were also detected within quail groups under unified conditions. Collectively, these findings indicate that poultry species acts as a critical factor affecting egg antibiotic residue depletion, independent of dosage and administration route effects.

Although ENR and DOX are prohibited during the laying period, their use prior to laying is not explicitly forbidden. However, insufficient elimination before the onset of lay may cause residual carry-over into eggs. Using the withdrawal periods estimated in our study, we calculated the theoretical safe medication windows. The calculation logic is as follows: subtracting the withdrawal period from the anticipated onset of lay gives the latest acceptable pre-laying age for completing the 5-day medication course, rather than for starting it. In practice, the entire 5-day treatment must be completed by this age to ensure that the withdrawal period is completed before the earliest possible onset of lay. The earliest age of first egg production for Chinese White-feathered quails was 35 days in our study. This age was adopted as the starting point of laying onset. The theoretical safe windows were calculated as follows: 24.25 days of age (high-dose ENR group), 25.97 days of age (low-dose ENR group), 9.44 days of age (high-dose DOX group), and 13.70 days of age (low-dose DOX group). In theory, completing the 5-day medication course before this window should ensure that residues in the first egg after laying onset do not exceed the MRLs. Conversely, if the course is completed after this window, residual carry-over into the laying phase may still occur even if the withdrawal period is strictly followed.

However, the estimation of the safe medication window has certain limitations. First, residue detection data presented noticeable variability, which may affect withdrawal time and window calculations. Although analytical methods and experimental conditions were strictly controlled, physiological fluctuations, stress responses, and individual differences in actual drug intake under ad libitum drinking water administration still remain as sources of variability that may narrow the theoretical window. Second, uncertainties in the onset of lay and pre-laying medication use also constrain window accuracy. Previous studies of chicken have confirmed that pre-laying medication may produce higher residue peaks and longer persistence than laying-period medication [25]. Moreover, given the irregularity in the onset of lay [32,33], the actual medication window may be shorter. In addition, complex clinical regimens such as combined or alternating drug use in practical production may add further uncertainty. Overall, while these theoretical windows should be interpreted cautiously and warrant further validation, they still provide a useful scientific reference for residue risk management in quail production.

The windows estimated in our study indicate that even under relatively ideal experimental conditions, the operational flexibility for ENR and DOX use is extremely limited. The window for DOX is less than 14 days of age, which makes it nearly impractical in actual production. Moreover, medication during the quail rearing phase is primarily used for preventive purposes. Immune schedules and disease outbreaks are unpredictable, and farmers face challenges in precisely timing therapeutic interventions within the narrow window. This situation increases the risk of residue non-compliance. Therefore, it is strongly recommended to minimize the use of ENR and DOX in egg-type quails production, with particular caution regarding DOX. If their use is deemed necessary for therapeutic purposes, administration should be strictly controlled in terms of timing and dosage under veterinary supervision, with full consideration of the residue elimination risks identified in this study.

## 5. Conclusions

This study investigated the residue depletion profiles of enrofloxacin (ENR) and doxycycline (DOX) in quail eggs following administration via drinking water. Residues of both drugs were higher in albumen than in yolk, with earlier peak times and more rapid decline in the albumen. ENR was present mainly as the parent compound and its metabolite ciprofloxacin (CIP), with CIP showing a delayed accumulation pattern. The withdrawal times for ENR were 10.75 days (high-dose group) and 9.03 days (low-dose group); for DOX, the corresponding values were 25.56 days and 21.30 days, respectively. Notably, DOX residues remained above the LOQ until 42 days after withdrawal. Based on these withdrawal times, the theoretical safe medication windows were estimated as 24.25 and 25.97 days of age for ENR, and 9.44 and 13.70 days of age for DOX. Notably, the theoretical safe medication window for DOX was less than 14 days, suggesting limited practical applicability in commercial egg-type quail production. Therefore, DOX should be prohibited for use in egg-type quails under this drinking water administration regimen. The estimated withdrawal times and theoretical safe medication windows in this study provide important references for regulatory authorities in establishing appropriate withdrawal intervals, for veterinary practitioners in exercising judicious drug use, and for competent authorities in conducting risk monitoring of quail egg products.

## Figures and Tables

**Table 1:** Grouping and design of residue depletion tests.

Group	I	Enrofloxacin		Doxycycline	
		II	III	IV	V
Administration dose	Control group	Low-dose 0.075 g/L ^1^	High-dose 0.15 g/L	Low-dose 0.15 g/L	High-dose 0.3 g/L

^1^ g/L: represents the drug amount dissolved in 1 L of water, expressed as active pharmaceutical ingredient.

**Table 2:** Characteristic ion parameters and mass-spectrometry conditions for target analytes.

Analytes	Retention Time (min)	Precursor Ion (*m*/*z*)	Product Ion (*m*/*z*)	Ion Type	Collision Energy (V)	Declustering Potential (V)
Enrofloxacin	4.09	360.0	316.1	Quantifier ion	25	80
			245.1	Qualifier ion	35	80
Ciprofloxacin	3.86	332.1	288.1	Quantifier ion	25	80
			245.1	Qualifier ion	33	80
Doxycycline	4.57	445.0	428.1	Quantifier ion	14	30
			154.1	Qualifier ion	20	30

**Table 3:** The evaluation results of the methodology.

		2 μg/kg			10 μg/kg			50 μg/kg					
Analytes	Matrix	Recovery (%)	Intra-Day RSD (%)	Inter-Day RSD (%)	Recovery (%)	Intra-Day RSD (%)	Inter-Day RSD (%)	Recovery (%)	Intra-Day RSD (%)	Inter-Day RSD (%)	R^2^	LOD	LOQ
ENR	Whole egg	90.65	4.9	5.2	92.71	5.3	5.8	96.78	4.8	4.7	0.99970	1	2
	Albumen	92.68	4.7	4.8	94.52	4.4	4.9	93.55	4.1	4.5	0.99877	1	2
	York	88.54	5.2	5.7	92.06	4.9	5.7	91.27	4.6	4.9	0.99930	1	2
CIP	Whole egg	97.65	6.5	6.8	91.76	6.2	6.4	95.13	6.1	6.8	0.99955	1	2
	Albumen	93.59	5.9	6.3	94.88	6	5.8	96.45	6	6.3	0.99764	1	2
	York	94.67	6.8	7	95.66	6.7	7.2	93.54	6.4	7.4	0.99787	1	2
DOX	Whole egg	76.27	6.9	7.6	80.33	6.2	6.5	89.33	5.7	6.4	0.99987	1	2
	Albumen	83.81	5.3	5.6	82.54	6.9	7.5	85.54	6.6	7.1	0.99812	1	2
	York	70.35	9.4	9.8	72.09	8.4	8.5	78.96	7.9	7.8	0.99932	1	2

## Data Availability

The raw data supporting the conclusions of this article will be made available by the authors on request.

## Supplementary Materials

The following supporting information can be downloaded at: https://www.mdpi.com/article/10.3390/vetsci13090917/s1, Figure S1: Standard curve for enrofloxacin matrix matching in quail eggs (2~100 ng/mL); Figure S2: Standard curve for ciprofloxacin matrix matching in quail eggs (2~100 ng/mL); Figure S3 Standard curve for doxycycline matrix matching in quail eggs (2~100 ng/mL); Table S1 Elimination parameters of DOX, ENR and CIP in quail eggs; Table S2: The results of enrofloxacin (ENR) and its metabolite ciprofloxacin (CIP) residue in quail eggs; Table S3: The results of doxycycline(DOX) residue in quail eggs.

## Abbreviations

The following abbreviations are used in this manuscript:

CIP	Ciprofloxacin
DOX	Doxycycline,
ENR	Enrofloxacin
EU	European Union
FDA	Food and Drug Administration
LC-MS/MS	Liquid Chromatography–Tandem Mass Spectrometry
LOD	Limit of detection
LOQ	Limit of quantification
MRLs	Maximum residue limits
USA	United States of America
