# Supplementary material for: Residue Depletion of Enrofloxacin and Doxycycline in Eggs of Chinese White-Feathered Quail After Administration via Drinking Water
Source: Vet Sci. 2026 Sep 7;13(9):917. doi: 10.3390/vetsci13090917 (PMC13612006; doi:10.3390/vetsci13090917)
Supplement: Supplementary file 1 [file vetsci-13-00917-s001.zip › vetsci-4501713-supplementary.pdf]

**Table S1** Elimination parameters of DOX, ENR and CIP in quail eggs

| | Analytes | Initial phases | | Terminal phases | |
|---|---|---|---|---|---|
| | | **K($d^{-1}$)** | **$T_{1/2}$(d)** | **K($d^{-1}$)** | **$T_{1/2}$(d)** |
| Low-dose | DOX | 0.37 | 1.86 | 0.13 | 5.04 |
| | ENR | 0.72 | 0.96 | 0.54 | 1.29 |
| | CIP | 0.64 | 1.08 | 0.49 | 1.4 |
| High-dose | DOX | 0.42 | 1.64 | 0.16 | 4.33 |
| | ENR | 0.76 | 0.91 | 0.4 | 1.73 |
| | CIP | 0.55 | 1.26 | 0.48 | 1.43 |

**Table S2** The results of Enrofloxacin (ENR) and its metabolite Ciprofloxacin (CIP) residue in quail eggs

μg/kg

| | | York | | | Albumen | | | Whole egg | | |
|---|---|---|---|---|---|---|---|---|---|---|
| | Time(d) | ENR | CIP | ENR+CIP | ENR | CIP | ENR+CIP | ENR | CIP | ENR+CIP |
| Low-Dose | T-1d | 20.91±4.23 | 4.68±2.55 | 25.59±6.13 | 298.66±40.69 | 20.61±3.71 | 319.27±37.51 | 150.38±16.32 | 8.24±1.33 | 158.62±16.79 |
| | T-2d | 824.46±110.46 | 37.64±14.96 | 862.1±106.92 | 1549.47±379.86 | 134.55±22.75 | 1684.02±376.90 | 1230.22±177.91 | 127.75±27.93 | 1357.97±219.56 |
| | T-3d | 1267.05±251.96 | 194.54±57.89 | 1461.59±247.04 | 1820.31±405.63 | 375.20±63.82 | 2195.51±388.17 | 1437.19±320.55 | 316.37±40.70 | 1753.56±430.23 |
| | T-4d | 1187.82±196.87 | 273.01±78.29 | 1460.83±203.93 | 1800.62±431.54 | 397.56±53.34 | 2198.18±413.41 | 1596.87±294.88 | 343.51±47.32 | 1940.38±186.30 |
| | T-5d | 1304.56±209.37 | 313.08±100.05 | 1617.64±325.60 | 2015.42±532.55 | 453.87±71.82 | 2469.30±566.38 | 1703.21±313.84 | 414.17±73.75 | 2117.39±430.50 |
| | D-1d | 1068.86±169.16 | 357.87±69.36 | 1426.73±172.60 | 779.66±129.16 | 352.32±60.10 | 1131.98±106.73 | 1062.37±167.82 | 354.83±83.31 | 1417.21±225.33 |
| | D-2d | 560.76±38.76 | 309.97±115.22 | 870.73±116.63 | 340.89±91.07 | 49.06±12.19 | 389.95±96.63 | 492.82±70.31 | 153.88±26.75 | 646.70±105.44 |
| | D-3d | 233.26±21.94 | 282.76±75.22 | 516.01±88.04 | 97.17±33.61 | 23.52±5.60 | 120.70±28.35 | 199.89±25.20 | 63.83±12.07 | 263.72±30.49 |
| | D-5d | 71.09±10.49 | 95.46±25.55 | 166.54±34.31 | 12.20±2.54 | 4.13±1.15 | 16.33±2.52 | 33.32±2.87 | 22.30±2.63 | 55.62±4.81 |
| | D-7d | 21.33±4.83 | 19.84±2.84 | 41.16±3.83 | 4.16±1.16 | 2.48±0.97 | 6.64±1.17 | 8.78±1.27 | 5.29±1.47 | 14.07±2.67 |
| | D-9d | 5.26±1.25 | 4.89±1.15 | 10.15±2.05 | 2.55±0.86 | 2.03±0.52 | 4.58±0.80 | 3.87±0.33 | 3.10±0.36 | 6.97±0.56 |
| | D-12d | NA | NA | NA | NA | NA | NA | NA | NA | NA |
| | D-15d | NA | NA | NA | NA | NA | NA | NA | NA | NA |
| | D-18d | NA | NA | NA | NA | NA | NA | NA | NA | NA |
| High-Dose | T-1d | 111.02±24.23 | 9.65±3.07 | 120.66±25.01 | 563.06±134.98 | 69.09±13.24 | 632.15±144.69 | 401.59±27.99 | 17.36±2.52 | 418.95±30.37 |
| | T-2d | 1446.13±344.59 | 69.99±15.67 | 1516.11±345.01 | 2288.23±403.73 | 290.22±81.87 | 2578.45±384.87 | 2098.46±236.27 | 209.43±51.88 | 2307.89±265.72 |
| | T-3d | 2221.10±436.89 | 326.75±77.61 | 2547.86±509.81 | 3258.69±609.77 | 638.76±114.33 | 3897.45±643.00 | 2825.28±272.99 | 437.83±102.06 | 3263.12±435.98 |
| | T-4d | 2587.08±350.27 | 475.14±120.36 | 3062.22±343.33 | 3459.08±492.39 | 594.63±202.09 | 4053.70±629.73 | 3074.69±347.96 | 492.23±126.45 | 3566.91±484.04 |
| | T-5d | 2742.31±243.38 | 603.60±163.55 | 3345.91±404.65 | 3767.05±385.82 | 784.63±191.24 | 4551.68±255.76 | 3306.67±475.02 | 697.78±115.91 | 4004.46±686.14 |
| | D-1d | 2218.55±270.57 | 582.82±119.91 | 2801.37±233.34 | 1549.61±303.03 | 675.69±200.99 | 2225.30±502.47 | 2047.93±242.04 | 653.82±95.83 | 2701.75±249.46 |
| | D-2d | 964.21±101.14 | 697.60±147.46 | 1661.81±101.85 | 592.71±113.02 | 152.52±36.97 | 745.23±101.61 | 849.49±99.76 | 385.18±59.52 | 1234.67±70.05 |
| | D-3d | 393.47±56.61 | 446.98±123.56 | 840.46±172.50 | 204.51±46.44 | 48.39±13.57 | 252.90±36.36 | 348.74±45.72 | 132.47±29.04 | 481.21±68.50 |
| | D-5d | 134.37±5.32 | 122.26±41.91 | 256.62±38.82 | 25.55±5.72 | 11.67±3.86 | 37.22±7.67 | 53.00±6.19 | 32.01±5.76 | 85.01±11.22 |

| | | | | | | | | | |
|---|---|---|---|---|---|---|---|---|---|
| D-7d | 54.17±10.31 | 48.18±8.63 | 102.35±17.41 | 11.18±2.94 | 3.21±0.83 | 12.39±3.42 | 20.86±1.71 | 11.35±2.87 | 32.21±2.68 |
| D-9d | 12.44±2.18 | 8.03±1.17 | 20.46±3.34 | 3.80±0.96 | 2.02±0.87 | 5.82±1.64 | 10.71±0.74 | 4.62±1.22 | 14.33±1.27 |
| D-12d | 3.69±0.65 | 2.47±0.36 | 6.16±0.52 | NA | NA | NA | 2.50±0.33 | NA | 2.50±0.33 |
| D-15d | NA | NA | NA | NA | NA | NA | NA | NA | NA |
| D-18d | NA | NA | NA | NA | NA | NA | NA | NA | NA |

Note: 1. NA indicates the detected value is below the LOQ; 2. T-1d denotes the first day of medication treatment, T-2d, T-3d... denote the second day, third day... of treatment; 3. D-1d denotes the first day of withdrawal, D-2d, D-3d... denote the second day, third day... of withdrawal, respectively.

**Table S3** The results of doxycycline(DOX) residue in quail eggs

μg/kg

| | Time(d) | Low-Dose | | | High-Dose | | |
|---|---|---|---|---|---|---|---|
| | | **whole egg** | **albumen** | **Yolk** | **whole egg** | **albumen** | **Yolk** |
| Medication treatmen Period | T-1d | 12.28±3.37 | 15.54±3.45 | 10.85±3.14 | 24.01±5.25 | 34.65±5.46 | 18.20±2.95 |
| | T-2d | 158.50±32.94 | 212.42±25.30 | 115.06±34.41 | 285.81±55.03 | 364.84±67.45 | 236.13±48.06 |
| | T-3d | 378.51±117.07 | 465.44±103.15 | 307.77±40.09 | 700.27±116.49 | 958.95±392.22 | 584.09±117.20 |
| | T-4d | 616.59±140.48 | 868.93±276.07 | 447.54±120.82 | 1592.82±556.20 | 1983.43±461.56 | 792.51±205.52 |
| | T-5d | 829.15±217.73 | 1028.26±333.85 | 511.31±81.99 | 1889.94±532.89 | 2428.53±603.42 | 1122.15±311.97 |
| Withdrawal time | D-1d | 768.09±189.92 | 772.57±148.77 | 675.41±147.67 | 1206.57±320.20 | 1347.54±290.92 | 1452.77±497.03 |
| | D-2d | 358.38±102.44 | 291.08±79.72 | 505.64±95.30 | 866.44±207.01 | 642.56±126.84 | 1120.12±293.88 |
| | D-3d | 177.70±51.80 | 151.62±36.58 | 342.77±76.57 | 530.59±118.54 | 375.98±48.90 | 794.65±143.24 |
| | D-5d | 164.11±43.81 | 21.87±8.27 | 176.91±33.25 | 228.51±43.97 | 53.73±7.75 | 360.09±81.40 |
| | D-9d | 74.65±19.81 | 11.52±1.35 | 112.44±17.31 | 142.93±13.56 | 28.66±5.27 | 230.22±28.43 |
| | D-15d | 18.44±2.52 | 6.38±1.08 | 43.98±11.27 | 44.92±6.40 | 9.29±1.61 | 99.53±20.61 |
| | D-21d | 3.91±0.66 | 2.07±0.69 | 11.64±2.68 | 10.74±2.09 | 3.11±0.73 | 22.53±5.44 |
| | D-28d | 2.71±0.25 | NA | 2.73±0.12 | 3.07±0.94 | 2.77±0.33 | 4.68±1.88 |
| | D-35d | 2.08±0.18 | NA | 2.33±0.21 | 2.86±0.47 | NA | 2.75±0.28 |
| | D-42d | NA | NA | NA | NA | NA | NA |
| | D-49d | NA | NA | NA | NA | NA | NA |
| | D-56d | NA | NA | NA | NA | NA | NA |

Note: 1. NA indicates the detected value is below the LOQ; 2. T-1d denotes the first day of medication treatment, T-2d, T-3d... denote the second day, third day... of treatment; 3. D-1d denotes the first day of withdrawal, D-2d, D-3d... denote the second day, third day... of withdrawal, respectively.

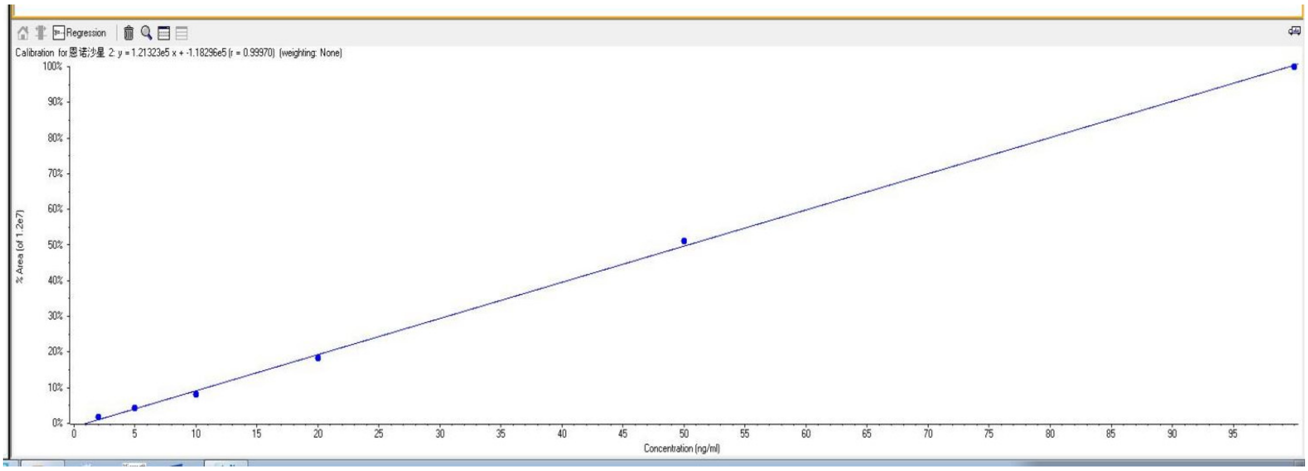


**Figure S1** Standard curve for enrofloxacin matrix matching in quail eggs (2～100ng/mL)

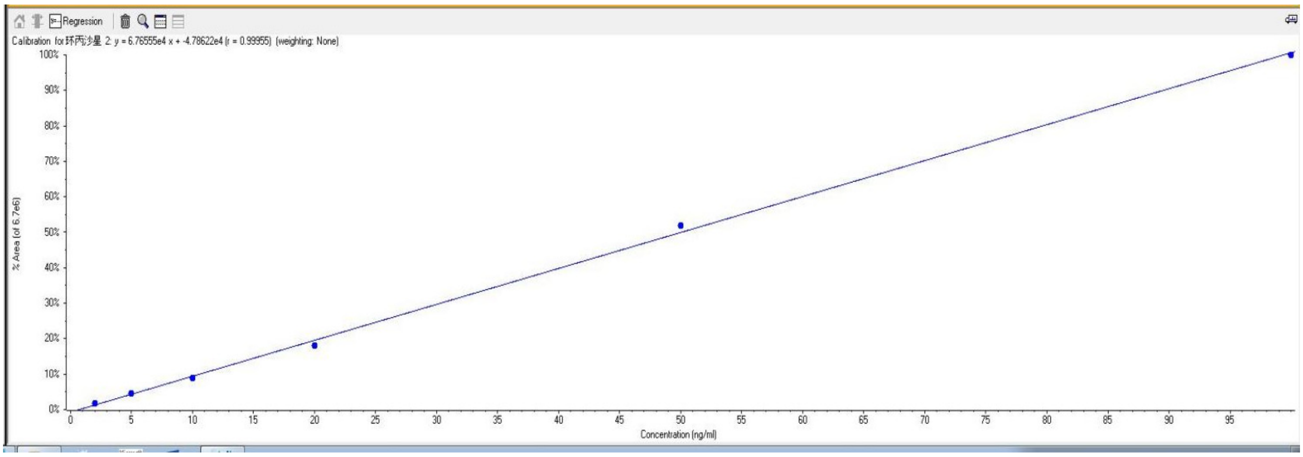


**Figure S2** Standard curve for ciprofloxacin matrix matching in quail eggs (2～100ng/mL)

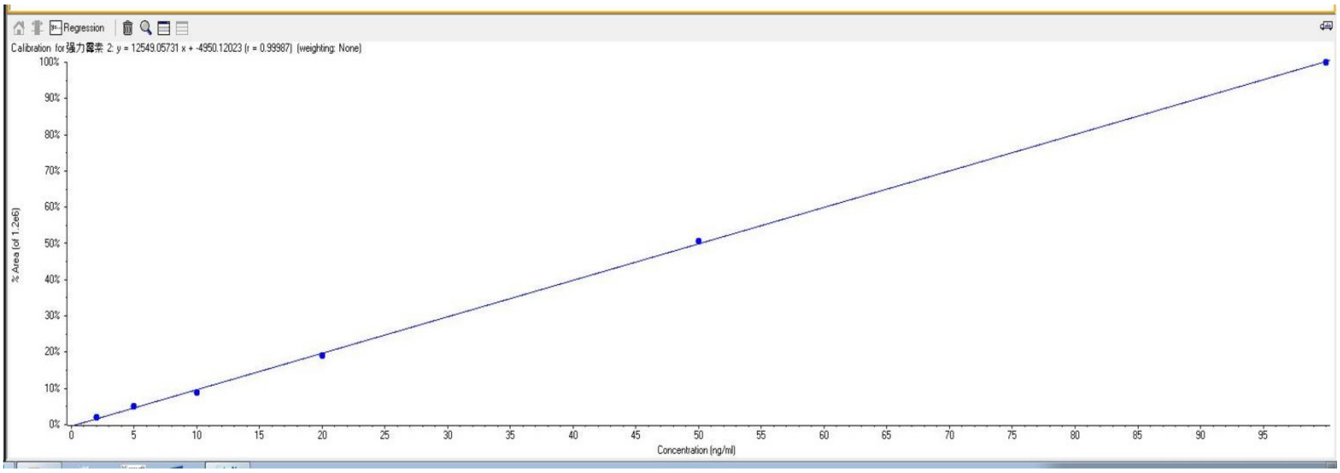


**Figure S3** Standard curve for doxycycline matrix matching in quail eggs (2～100ng/mL)
